# P-1606. Clearance of Infectious Virus in COVID-19 Patients Treated with Ensitrelvir Across Vaccination Status, Age, and Risk Subgroups: Exploratory Analyses from the SCORPIO-HR Study

**DOI:** 10.1093/ofid/ofaf695.1785

**Published:** 2026-01-11

**Authors:** Florin Draica, Annie Luetkemeyer, Kara W Chew, Judith S Currier, David Smith, Simon Portsmouth, Aeron C Hurt, Takahiro Hasegawa, Safwan Kezbor, Daniel Verdi, Takeki Uehara

**Affiliations:** Medical Affairs, Shionogi, Inc., Florham Park, NJ, USA, Florham Park, NJ; University of California San Francisco, San Francisco, CA; David Geffen School of Medicine at University of California, Los Angeles, California; David Geffen School of Medicine at University of California, Los Angeles, California; University of California, San Diego, San Diego, California; Shionogi Inc, Florham Park, NJ; Clinical Development, Shionogi & Co., Ltd., London, England, UK, London, England, United Kingdom; SHIONOGI & CO., LTD., Osaka-shi, Osaka, Japan; Clinical Development, Shionogi Inc., Florham Park, NJ, USA, Florham Park, New Jersey; Medical Affairs, Shionogi, Inc., Florham Park, NJ, USA, Florham Park, NJ; SHIONOGI & CO., LTD., Osaka-shi, Osaka, Japan

## Abstract

**Background:**

Many antiviral studies for COVID-19 have utilized molecular methods to assess viral load; however, viral RNA may remain detectable even after the infectious virus is cleared. Viral culture offers a direct measure of active viral shedding, with implications for transmission.

**Figure:**
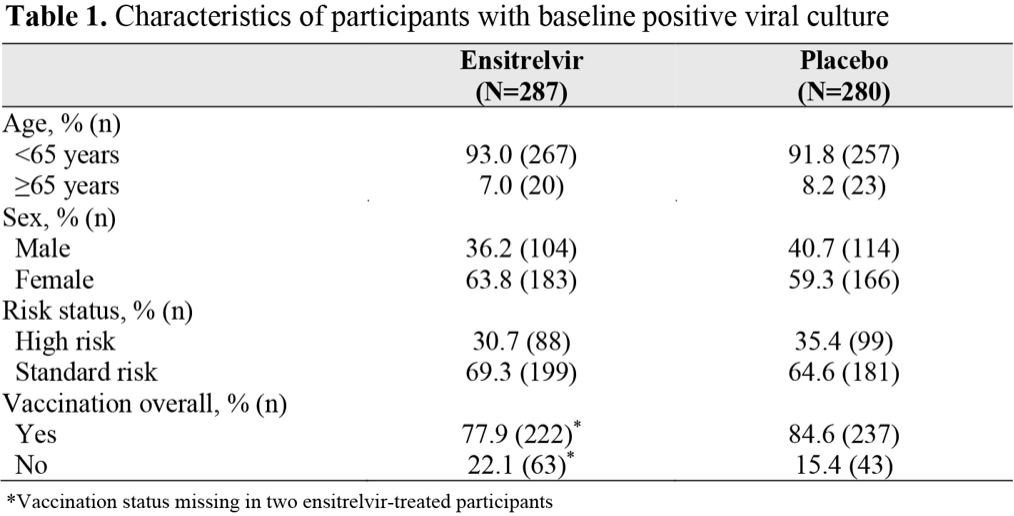

**Figure:**
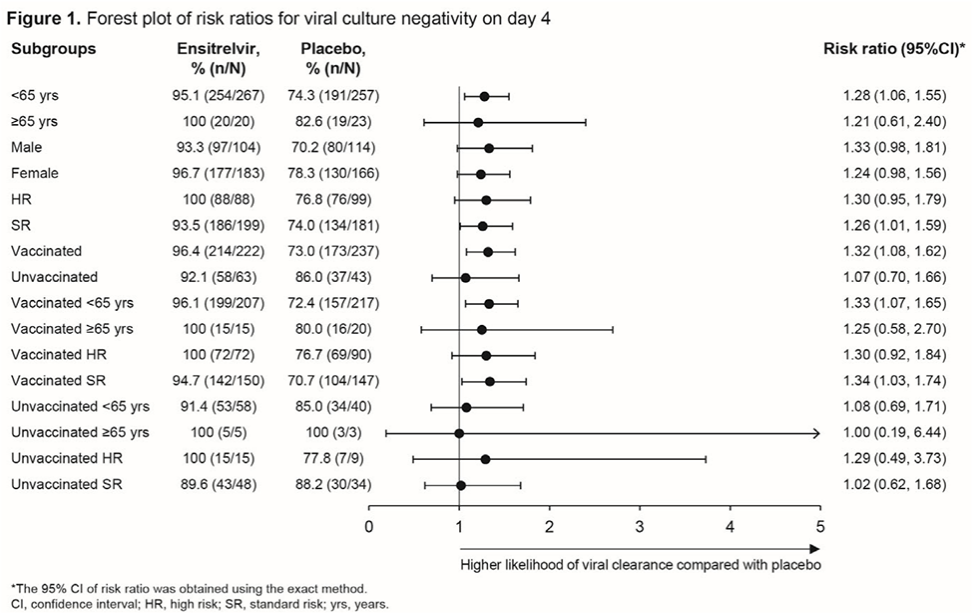

**Methods:**

This exploratory analysis of the phase 3 SCORPIO-HR treatment study evaluated the effect of ensitrelvir versus placebo on the clearance of infectious virus on day 4 in patients with baseline positive viral culture, stratified by risk status for severe COVID-19 (high risk, standard risk), age (< 65 years, ≥ 65 years), and COVID-19 vaccination status (vaccinated, unvaccinated) (Table 1). Viral culture data in these subgroups are presented for ensitrelvir- versus placebo-treated patients. The endpoint in this exploratory analysis was the proportion of participants with negative SARS-CoV-2 viral culture on day 4.

**Results:**

Among participants with positive viral cultures at enrollment (28.7% [601/2093]), 95.5% (274/287) of ensitrelvir-treated and 75.0% (210/280) of placebo-treated participants achieved viral culture negativity by day 4. Across all subgroups, more patients in the ensitrelvir group achieved negative viral culture than those in the placebo group. Among vaccinated participants, viral culture clearance was consistently higher with ensitrelvir than with placebo, regardless of risk status and age. By day 4, patients < 65 years of age who were vaccinated and categorized as standard risk showed a significantly higher likelihood of achieving viral culture negativity when treated with ensitrelvir compared with placebo (Figure 1).

**Conclusion:**

Ensitrelvir demonstrated consistent antiviral efficacy, as measured by viral culture clearance, irrespective of participants’ baseline characteristics, including vaccination status, age, and risk status for severe disease.

**Disclosures:**

Florin Draica, MD, CMD, MBA, Shionogi, Inc.: Employee Annie Luetkemeyer, MD, Cepheid: Grant/Research Support|Gilead: Grant/Research Support|GSK: Grant/Research Support|Merck: Grant/Research Support|ViiV: Grant/Research Support Kara W. Chew, M.D., M.S., Pfizer: Medical writing support (no direct compensation) Judith S. Currier, MD, MSc, Merck and Co.: Honoraria David Smith, MD, MAS, Bayer: Advisor/Consultant|Biosciences: Advisor/Consultant|Fluxergy: Stocks/Bonds (Private Company)|Gilead: Advisor/Consultant|Hyundai: Advisor/Consultant|IAS USA: Honoraria|Linear Therapies: Stocks/Bonds (Private Company)|Model Medicines: Advisor/Consultant|Model Medicines: Stocks/Bonds (Private Company)|NIH: Grant/Research Support|Pharma Holdings: Advisor/Consultant|Red Queen Therapeutics: Advisor/Consultant Simon Portsmouth, MD, Shionogi Inc.: Employee Aeron C. Hurt, Ph.D., Shionogi, Inc.: Employee Takahiro Hasegawa, DrPH, Shionogi, Inc.: Employee Safwan Kezbor, M.D., Shionogi, Inc.: Employee Daniel Verdi, M.D., Shionogi, Inc.: Employee Takeki Uehara, Ph.D., Shionogi, Inc.: Employee

